# *NR3C1* hypermethylation in depressed and bullied adolescents

**DOI:** 10.1038/s41398-018-0169-8

**Published:** 2018-06-19

**Authors:** Paschalis Efstathopoulos, Filip Andersson, Philippe A. Melas, Liu L. Yang, J. Carlos Villaescusa, Joëlle Rȕegg, Tomas J. Ekström, Yvonne Forsell, Maria Rosaria Galanti, Catharina Lavebratt

**Affiliations:** 1 Department of Molecular Medicine and Surgery, Karolinska Institutet, Stockholm, Sweden; 2 Center for Molecular Medicine, Karolinska University Hospital, Stockholm, Sweden; 3 Department of Public Health Sciences, Karolinska Institutet, Solna, Sweden; 4 Department of Clinical Neuroscience, Karolinska Institutet, Stockholm, Sweden; 5 Swetox, Unit of Toxicology Sciences, Karolinska Institutet, Stockholm, Sweden; 6 Centre for Epidemiology and Community Medicine, Stockholm County Council, Stockholm, Sweden

## Abstract

The disruption of key epigenetic processes during critical periods of brain development can increase an individual’s vulnerability to psychopathology later in life. For instance, DNA methylation in the glucocorticoid receptor gene (*NR3C1*) in adulthood is known to be associated with early-life adversities and has been suggested to mediate the development of stress-related disorders. However, the association between *NR3C1* methylation and the emergence of internalizing symptoms in childhood and adolescence has not been studied extensively. In the present report, we used saliva DNA from a cohort of Swedish adolescents (13–14 years old; *N* = 1149) to measure *NR3C1* methylation in the exon 1F region. Internalizing psychopathological symptoms were assessed using the Center for Epidemiologic Studies Depression Scale for Children (CES-DC). We found that *NR3C1* hypermethylation was cross-sectionally associated with high score for internalizing symptoms in the whole group as well as among the female participants. In addition, an analysis of social environmental stressors revealed that reports of bullied or lacking friends were significantly associated with *NR3C1* hypermethylation. This cross-sectional association of *NR3C1* exon 1F hypermethylation with internalizing psychopathology in adolescents, as well as with bullying and lack of friends are novel results in this field. Longitudinal studies are needed to address whether *NR3C1* methylation mediates the link between social stressors and psychopathology in adolescence.

## Introduction

Adolescence represents a transitional state between childhood and adulthood that is accompanied both by marked endogenous changes, e.g., pubertal increases in hormonal levels, and by increased demand from the surrounding environment, e.g., social and intellectual performances. Adolescence also constitutes a critical period for brain maturation and is characterized by heightened responsiveness to incentives and socioemotional stimuli^1^. Anxiety and affective disorders often have their onset or prodromal phase in adolescence^2^.

Chronic stress is a risk factor for anxiety and affective disorders. Stress refers to the innate response of an organism to environmental threats that allows for physiological and behavioral adaptations in order to maintain homeostasis. In mammals, this response is mainly mediated through the activation of the hypothalamic–pituitary axis in the brain, which results in the secretion of cortisol from the adrenal glands; collectively forming the hypothalamic–pituitary–adrenal (HPA) axis stress response system. Although this cascade of events initially increases awareness and behavioral flexibility^3^, disturbances in the regulation of the HPA axis during stress can result in the development of psychopathology^4^. For example, internalizing disorders, including anxiety and depression, have been associated with increased cortisol secretion and a hyper-active HPA axis^5,6^

On the cellular level, cortisol acts by binding to the glucocorticoid receptor (GR), a pleiotropic transcription factor that is encoded by the *NR3C1* gene. Upon binding to GR, cortisol induces a number of stress-related responses to acute and chronic stress. Yet, one of GR’s critical roles is to mediate negative feedback regulation of the HPA axis to terminate the stress response. Various mechanisms can reduce the sensitivity of this negative feedback loop resulting in sustained high cortisol levels. One such mechanism is the decrease in *NR3C1* expression by DNA methylation. DNA methylation is an epigenetic mechanism occurring mainly at cytosine residues at CpG sites. *NR3C1* contains nine untranslated alternative exon 1 variants^7^ that are independently controlled by unique upstream promoters^8^. Most of these alternative first exons, including their promoters, are found within a sequence region rich of CpGs. Upon methylation, CpG-containing sequence sites can no longer serve as binding sites for their transcription factors and, consequently, the mRNA and protein expression of *NR3C1* is reduced^9^.

Preclinical studies have demonstrated that early environmental stressors, e.g., lack of maternal care, can cause increased methylation (hypermethylation) of specific CpG regions and alter the HPA axis responses to stress^10^. Early-life traumas in humans, in the form of childhood abuse or parental loss, were also found in adults to be associated with *NR3C1* hypermethylation in CpGs of the exon 1F region, which includes a binding site of the transcription factor NGFI-A^11–15^. Hypermethylation of the same exon was also found in infants of depressed mothers^16,17^. Furthermore, there are studies showing that childhood abuse is associated with DNA hypermethylation in adults in other exon 1 variants of *NR3C1*, including exons 1B, 1C, and 1H^9^. On the other hand, a recent human study reported hypomethylation of *NR3C1* exon 1F region in the case of early adversity and the presence of substance abuse, depressive or anxiety disorders^18^; these findings suggest complexity in the interaction between adversity exposure and the DNA methylation of this gene.

Despite the extensive literature on *NR3C1* methylation as mediator of the effects of retrospective early adversities on the development of psychopathology later in life^19,20^, less is known about how *NR3C1* methylation is associated with recent or current adversities and psychopathology, especially during adolescence. In the few available studies, *NR3C1* exon 1F hypermethylation was reported to be associated with internalizing symptoms in children at age 4–16 years^21^, and in another study, *NR3C1* exon 1H and 1F hypermethylation was reported to be associated with traumatic youth experiences and milder stressful life events in adolescents aged 16–18 years^22^. Given the limited literature focusing on adolescents, we measured DNA methylation levels of *NR3C1*’s exon 1F in a sample of adolescent individuals, aged 13–14 years old, and investigated the cross-sectional associations between methylation and internalizing psychopathological symptoms on the one side; and between methylation and exposure to environmental stressors on the other side.

The study hypotheses were as follows: *NR3C1* exon 1F methylation would be higher among adolescents with internalizing symptomatology than among those without these symptoms. Since exon 1F methylation level was previously reported to be positively associated with total stress-induced cortisol output in adult healthy women but not in men^23^, and peripheral blood cortisol level is positively associated with internalizing disorder^24^, sex stratified analyses were performed. In line with the previous evidence of increased *NR3C1* exon 1F methylation in those exposed to early maltreatment^19^ we hypothesized that *NR3C1* exon 1F hypermethylation would be higher among adolescents reporting social stressors (e.g., bullying that was previously reported to be associated with depressive psychopathology^25^) than among those without this experience.

## Materials and methods

### Study participants

Study participants consisted of children attending the seventh grade (13–14 years old) of compulsory school in both urban and rural areas of southern and central Sweden. The participant recruitment took place during the school years fall 2013–spring 2014 and fall 2014–spring 2015 as part of the KUPOL project^26^. For this project, a total of 3959 children, from 101 schools, and their legal guardians gave informed consent and answered the study questionnaires. Saliva samples were collected from a random subsample of 1315 consenting students, of which 1149 were successfully analyzed (Supplementary figure). Complete demographics, and detailed information on the KUPOL project, have already been published elsewhere^26^. The study was approved by the Stockholm Ethics Review Board, and written informed consent was obtained from all participants.

### Assessment of internalizing symptoms

Internalizing symptoms were assessed by means of the Center for Epidemiologic Studies Depression Scale for Children (CES-DC), which evaluates symptoms of depression and anxiety (referred as internalizing symptoms), and was self-reported by the students. The score threshold for presence (yes/no) of symptoms was CES-DC ≥ 30, which has demonstrated high specificity in Swedish population samples^27^.

### Psycho-social stressors

We considered some psycho-social events or conditions as predictors of epigenetic changes (*NR3C1* hypermethylation). All information on these factors was self-reported in questionnaires either by the students (being bullied, lack of friends, alcohol and tobacco use, and cohabitation with parents) or by their parents (parental education, employment status, and birth country).

### DNA sample preparation

Saliva samples (4 ml) were collected from the participants using a whole-saliva collection device (Oragene•DNA; DNA Genotek Inc., Ottawa, Canada). DNA was successfully extracted from 1304 saliva samples using a protocol that is based on magnetic bead separation according to chemagen technology (PerkinElmer) on a ChemagicStar^®^-robot (Hamilton), and was dissolved in 500 µl 10 mM Tris-HCl buffer, pH 8.0. DNA concentration and purity were measured by ultraviolet absorbance.

### DNA methylation analysis

**Fig. 1:**
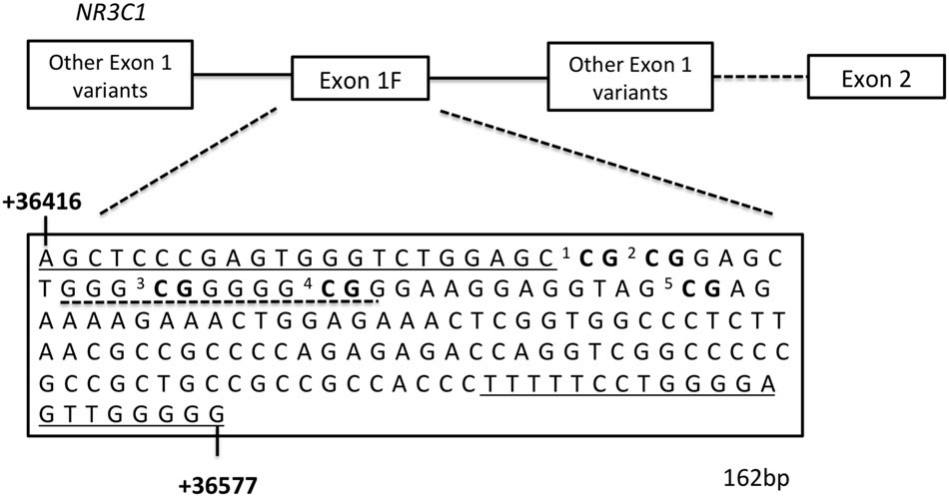
The *NR3C1* exon 1F sequence showing the CpG sites CpG1–5 (bold) whose DNA methylation levels were quantified in the present study. The CpG sites CpG1–5 correspond to the CpGs 35–39 analyzed in McGowan et al.^11^ and Melas et al.^12^. The broken underlined sequence corresponds to nerve growth factor-induced protein A (NGFI-A) binding site, as reported by McGowan et al.^11^. The solid underlined sequences correspond to the primers

Bisulfite conversion of the DNA was performed using EZ-96 DNA Methylation-Gold™ MagPrep kit (Zymo Research Corporation; Irvine; USA) according to the manufacturer’s protocol and the processed DNA was stored at −20 °C until further analysis. A 162-base pair (bp) fragment, corresponding to the *NR3C1* exon 1F region (NCBI reference sequence: NG_009062.1 (+36 416 to +36 577) from the transcriptional start site), was amplified using the PyroMark PCR Kit (Qiagen, Hilden, Germany) and the following primers: forward 5′-AGTTTTAGAGTGGGTTTGGAG-3′, reverse biotin-5′-CCCCCAACTCCCCAAAAA-3′. PCR conditions were as follows: 94 °C for 15 min, followed by 45 cycles of 94 °C for 30 s, 60 °C for 60 s and 72 °C for 30 s with a final extension of 10 min at 72 °C. For pyrosequencing the sequencing primer, 5′-GAGTGGGTTTGGAGT-3′, was used to sequence a 50 bp region, of the 162 bp PCR fragment, containing five CpG sites, including the NGFI-A-binding site at CpG3 (Fig. 1). Pyrosequencing was performed on a Pyromark Q96 device (Qiagen, Hilden, Germany) and data were analyzed using the Pyromark Q96 5.2.8 software. DNA methylation data that passed quality control for all CpGs were obtained from 1184 out of the 1304 samples, and used for subsequent analyses. The average between-plate coefficient of variation for CpG1–5 was estimated at 10.3% (range 4.5–16.2%) based on the following control samples prepared in duplicates: 0, 6, 9, and 12% methylated DNA controls, prepared by combining different dilutions of commercially available fully methylated and non-methylated genomic human HCT116 DKO DNA (Zymo Research Corporation). Ninety-eight percent of the samples were within 0–12% methylation at any of the CpG sites studied. The analyses were performed blinded to the phenotypes.

### Statistical analyses

The association between *NR3C1* methylation and internalizing symptoms was assessed on a univariate level for all CpG sites separately. For all models the outcome was a binary indicator (yes/no) of symptoms on the individual level of the participant and the explanatory covariate was one of the CpGs. Due to the high number of participants having 0% methylation at a CpG site, data were categorized for each CpG into three groups, as follows: (1) unmethylated group: participants with no detectable methylation (0%); (2) low methylation group: methylation levels below the median of detectable methylation; and (3) high methylation group: methylation levels above the aforementioned median. Logistic regression analyses, with or without sex stratification, were performed with regard to the presence of internalizing symptoms according to the CES-DC scale, dichotomized at a cutoff of ≥30. In all analyses the unmethylated group constituted the reference. A sensitivity analysis was performed to estimate the probability of false positive CES-DC score vs methylation level score associations. We performed 10 000 permutations of random rearrangement of the links between CES-DC score and methylation level score. For each of the 10 000 simulations we calculated the association of methylation on depression, where after we compared the distribution of the simulated outcomes vs the actual outcomes from our data. In order to examine associations between *NR3C1* methylation and potential stressors in the social environment, we performed regression analyses where the following covariates constituted the predictors of methylation level: experience of being bullied; lack of friends in school; current use of tobacco; use of alcohol during the past year; both parents with education below university level; at least one parent unemployed; at least one parent born outside Sweden; and parents not living together. In this analysis the outcome was *NR3C1* methylation (low or high methylated vs unmethylated). Sex-specific analyses were not performed due to the limited sample size. All data management and statistical analyses were performed using Stata 14.2. All test assumptions were fulfilled. All statistical tests are two-sided.

## Results

### Prevalence of psychopathological symptoms

The analytical sample comprised 1149 grade 7 school children from 101 schools, i.e., those children recruited in the cohort who provided DNA samples that passed quality control and answered the CES-DC scale for the assessment of the psychopathological internalizing symptoms (Supplementary Figure). The prevalence of symptoms above the chosen cutoff was 13.8% in females and 2.5% in males (Table 1). A comparison of symptom prevalence between all participating adolescents and those in the sample included in this study showed very similar estimates (Table 1).

**Table 1:** Prevalence of depressive psychopathological symptoms, by sex

	All males *N* = 1933	All females *N* = 2026	Males with DNA samples *N* = 524	Females with DNA samples *N* = 625
CES-DC (depression-anxiety)				
Above threshold %	2.5	14.4	2.5	13.8
Below threshold %	86.5	77.1	97.5	86.2
Missing %	11.0	8.5	—	—

The Kupol Study, Stockholm 2013–2015

*CES-DC* Center for Epidemiologic Studies Depression Scale for Children

### Association between *NR3C1* methylation and CES-DC internalizing symptoms

Eight percent to 51% of the sample showed methylation above 0% at the five CpG sites studied. The level of methylation (percent of DNA being methylated) in these samples was for the majority low, with medians spanning between 3 and 5% (Table 2). Since the distribution of methylation levels in our sample was very skewed toward zero we categorized it into three groups, the unmethylated group, the low methylation group with values between 0% and the median, and the high methylation group with values above the median. The mean and range of methylation level for each group and CpG site is presented in Supplementary Table S1. The *NR3C1* high methylation group was significantly associated with internalizing symptoms, for CpG sites 1, 2, and 3 (odds ratio (OR) range: 1.93–2.46; Table 3). A separate analysis by sex showed that these associations were detected only in females (point-wise OR range: 2.06–2.70; Table 3). Any methylation at the CpG5 site had a tendency for association with depression symptoms among females, but the estimates were not formally statistically significant. The prevalence of internalizing psychopathological symptoms in boys was too low to detect possible sex differences in association to *NR3C1* methylation.

**Table 2:** *NR3C1* methylation prevalence and group cutoffs

CpG site	Number (%) of saliva samples with methylation > 0% for each CpG		Median methylation level (% DNA being methylated) among the samples with methylation > 0%^a^	
	Males *N* = 524	Females *N* = 625	Males	Females
CpG1	87 (16.6)	118 (18.9)	3.1	3.9
CpG2	64 (12.2)	79 (12.6)	3.4	3.7
CpG3	261 (49.8)	319 (51.0)	3.6	3.4
CpG4	107 (20.4)	109 (17.4)	3.2	3.7
CpG5	40 (7.6)	56 (9.0)	4.3	4.8

The Kupol Study, Stockholm 2013–2015

^a^Medians used for categorizing an individual into low methylation group or high methylation group

**Table 3:** Cross-sectional odd ratios (OR) and corresponding 95% confidence interval (CI) of CES-DC score above the threshold, indicating depression symptoms, according to *NR3C1* methylation

Methylation site, category	All *N* = 1149		Males *N* = 524		Females *N* = 625	
	OR	95% CI	OR	95% CI	OR	95% CI
CpG1 low	1.47	0.75–2.87	0.72	0.09–5.68	1.77	0.84–3.70
CpG1 high	2.46	1.38–4.37	ND	ND	2.70	1.45–4.98
CpG2 low	1.23	0.55–2.77	1.10	0.34–8.70	1.34	0.54–3.32
CpG2 high	2.09	1.06–4.14	ND	ND	2.45	1.78–5.10
CpG3 low	1.09	0.63–1.87	0.69	0.14–3.47	1.12	0.62–2.01
CpG3 high	1.93	1.20–3.09	1.65	0.49–5.50	2.06	1.22–3.50
CpG4 low	1.45	0.57–2.29	1.45	0.31–6.81	1.31	0.59–2.91
CpG4 high	1.38	0.72–2.62	0.85	0.11–6.77	1.50	0.75–3.04
CpG5 low	1.94	0.85–4.47	ND	ND	2.42	0.99–5.92
CpG5 high	1.94	0.85–4.47	ND	ND	2.20	0.91–5.33

The Kupol Study, Stockholm 2013–2015

Reference category = unmethylated

*ND* not determined due to too few observations

In order to rule out the probability that we pick up false positive associations in our dataset, and that separation into low and high methylation groups was relevant for the type of analysis we did, we performed a sensitivity analysis with 10 000 permutations of random rearrangement of the links between CES-DC score and methylation level score. For each of the 10 000 simulations we calculated the association of methylation on development of internalizing symptoms. Next, we compared the distribution of the simulated outcomes vs the actual outcomes from our data. The results clearly showed that the associations of high methylation in CpG1 and CpG3 with CES-DC were very unlikely to be false positive, with estimates lying well above the 95th percentile of the distribution of the simulated estimates. Also the high methylation for CpG2 had results above the 95th percentile, albeit not as strong as the other two.

### *NR3C1* hypermethylation and potential social stressors

In order to explore the associations between *NR3C1* methylation level and potential childhood environmental stressors we analyzed level of methylation at CpG1–5 and selected covariates indicative of childhood stress measured in the KUPOL study. The mean and range of methylation level for each group and CpG site are presented in Table S1. The analysis of *NR3C1* methylation and putative environmental stressors showed that high methylation for CpG2 was significantly cross-sectionally associated with having been bullied or lack of friends (bullying: OR = 1.89, 95% confidence interval (CI) = 1.08–3.32; lack of friends: OR = 2.30, 95% CI = 1.09–4.86; Table 4). Although not statistically significant, the CpG sites 1, 4, and 5, also showed high methylation among those having been bullied (CpG1: OR = 1.26, 95% CI = 0.74–2.15; CpG4: OR = 1.24, 95% CI = 0.75–2.07; CpG5: OR = 1.79, 95% CI = 0.91–3.52; Table S4) and the CpG1 site showed high methylation among those lacking friends (CpG1: OR = 1.25, 95% CI = 0.58–2.70; Table S4). Means and range of methylation level for each CpG for bullied/not bullied as well as lacking friends/having friends are presented in Tables S2 and S3. None of the other putative stressors was associated with *NR3C1* methylation at any site.

**Table 4:** Cross-sectional odds ratio (OR) and corresponding 95% confidence interval of *NR3C1* CpG site 2 methylation according to potential stressors

Potential stressors	Proportion Non-methylated (%)	Proportion Low methylated (%)	OR_low vs zero_, 95% CI	Proportion High methylated (%)	OR_high vs zero_, 95% CI
Bullying					
Yes * N* = 180	83.3	6.7	1.11, 0.59–2.12	10.0	1.89, 1.08–3.32
No * N* = 949	88.1	6.3	Ref	5.6	Ref
Friends in school					
No * N* = 74	78.7	9.3	1.76, 0.77–4.02	12.0	2.30, 1.09–4.86
Yes * N* = 1061	88.2	5.9	Ref	5.9	Ref
Currently using tobacco					
Yes * N* = 15	93.3	6.7	0.99, 0.13–7.70	0.0	ND
No * N* = 1134	87.5	6.3	Ref	6.2	Ref
Alcohol the past year					
Yes * N* = 21	85.7	14.3	2.39, 0.69–8.34	0.0	ND
No * N* = 1117	87.6	6.1	Ref	6.3	Ref
Both parents’ education below university					
Yes * N* = 315	87.0	5.7	0.92, 0.53–1.60	7.3	1.30, 0.77–2.18
No * N* = 827	88.0	6.3	Ref	5.7	Ref
At least one parent unemployed					
Yes * N* = 196	88.8	4.1	0.62, 0.29–1.31	7.1	1.20, 0.65–2.21
No * N* = 936	87.6	6.5	Ref	5.9	Ref
At least one parent born outside Sweden					
Yes * N* = 196	88.3	5.1	0.80, 0.40–1.60	6.6	1.08, 0.58–2.02
No * N* = 903	87.6	6.3	Ref	6.1	Ref
Parents not cohabiting					
Yes * N* = 199	87.5	6.5	1.08, 0.58–2.02	6.0	1.00, 0.52–1.90
No * N* = 923	87.9	6.1	Ref	6.0	Ref

The Kupol Study, Stockholm 2013–2015

## Discussion

The DNA methylation status of the GR gene, *NR3C1*, specifically hypermethylation of the human exon 1F, has in adults proved to be associated with retrospective traumatic events in childhood, including emotional, physical, or sexual abuse; neglect; early parental death; and other traumatic events^11–13,15^, reviewed in refs. ^19,20^. Methylation levels and effect sizes are mostly consistent between these studies also when results from brain tissue and peripheral tissue such as leukocytes and lymphocytes were compared. The latter was proposed to possibly reflect a stress response signal acting coordinately on multiple cell types^19^. *NR3C1* exon 1F hypermethylation has been reported to also mediate the association between traumatic events and internalizing symptoms in preschoolers^28^ and to mediate an association between early adversity and borderline personality disorder in adults^29^. However, few studies have examined if *NR3C1* methylation is associated with recent or current adversities or with psychopathology during adolescence.

In the present report, we investigated the association of *NR3C1* methylation with depressive symptoms using a large sample of 13- to 14-year-old students in Sweden. The CpG sites that we analyzed are part of the *NR3C1* exon 1F and two are within the canonical binding site of the transcription factor NGFI-A (CpG3 and CpG4). We found that hypermethylation of *NR3C1*, at CpG1, 2, and 3, was associated with self-reported internalizing symptoms in adolescents. Interestingly, in preschoolers hypermethylation at exon 1F CpG3 represented the strongest association with internalizing symptoms^28^ and hypermethylation at a CpG of a neighboring NGFI-A-binding site associated with internalizing symptoms in 4- to 16-year-old kids^21^. In previous studies of adults, hypermethylated exon 1F CpGs associated with retrospective early adversity has also commonly been located in a putative NGFI-A-binding site, reviewed by Palma-Gudiel et al.^20^. The low methylation levels reported for the exon 1F CpGs is in agreement with them being located in a strong promoter region. The associations in our study remained significant when we analyzed only the females. The prevalence of internalizing psychopathological symptoms in boys was too low to detect possible sex differences in association to *NR3C1* methylation. However, sex differences in biological stress response pathways and cellular environment (e.g., sex hormones) may account for possible differences in *NR3C1* hypermethylation as well as the different prevalence of depressive psychopathology^23,30,31^.

Finally, in order to generate new hypotheses on factors in the social environment that could induce the methylation of *NR3C1*, we performed an analysis of potential social and behavioral stressors. Adolescents reporting lack of friends and those reporting having been bullied were significantly more likely to harbor *NR3C1* hypermethylation in CpG2, while the observed increase in methylation at CpG1, 4, and 5 was not statistically significant. Previously it was reported from adolescents aged 16–18 years that sexual abuse was associated with hypermethylation in exon 1F and 1H, whereas other life-threatening childhood or adolescence traumatic events associated with hypermethylation in exon 1H only, and milder stressful life events less strongly so^22^. A very recent study of genome-wide DNA methylation in relation to youth victimization in 1669 young persons, that is 50% more individuals than in our study, analyzed bullying but detected no genome-wide significant signal for bullying. Further, none of the 42 CpGs in *NR3C1* studied passed gene-wide significance threshold for victimization^32^, indicating complexity and limited effect size in the influence of adversity exposure on the *NR3C1* methylation, and possibly technical difficulties in analysis of low methylation levels.

This study had a number of limitations, the major one being the assessment of *NR3C1* methylation using DNA from a peripheral tissue (saliva DNA). Still, previous studies on *NR3C1* exon 1F methylation and early trauma have generated similar results by utilizing DNA from brain^10,11^, saliva^12,18,28^, and whole blood leukocytes^13,14,16,22^. In addition, the cross-sectional design prevented the study of the timing of methylation in relation to the timing of the stressful events and of the onset of symptoms. Therefore, the possibility that *NR3C1* methylation preceded these events cannot be ruled out. We also lacked information about traumatic events occurring earlier in the lives of these individuals, some of which may have caused symptoms, rather than later occurring events as it was suggested from an analysis of maltreated children at age 5^28^.To this end, longitudinal studies are needed in order to assess causal effects of stressful life events during adolescence on development of psychopathology later in life, as well as the possible mediator role of *NR3C1* methylation in this process.

In summary, this study reports the novel finding that hypermethylation of *NR3C1*’s exon 1F is cross-sectionally associated with internalizing symptoms in a large population of early adolescent females. Moreover, bullying and lack of friends during early adolescence was associated with higher *NR3C1* methylation levels, which supports the hypothesis of a molecular link between these types of stressors and internalizing psychopathological symptoms.

## Electronic supplementary material

Supplementary tables 1-4

Supplementary figure
