# Supplementary material for: NR3C1 hypermethylation in depressed and bullied adolescents
Source: Transl Psychiatry. 2018 Jun 19;8:121. doi: 10.1038/s41398-018-0169-8 (PMC6008402; doi:10.1038/s41398-018-0169-8)
Supplement: Supplementary file 1 — Supplementary tables 1-4 [file 41398_2018_169_MOESM1_ESM.docx]

**Supplementary Table S1.** Methylation level distribution for *NR3C1* CpG1, CpG3, CpG4 and CpG5 in the Low methylation group and the High methylation group. The Kupol Study, Stockholm 2013-2015.

| **CpG site** | **Methylation level for the samples with methylation > 0% and below median** | | **Methylation level for the samples with methylation above median** | |
| --- | --- | --- | --- | --- |
| **Total number of samples**  **N = 1149** | Mean | Range [min;max] | Mean | Range [min;max] |
| CpG1 | 2.64 | [1.15;3.58] | 7.40 | [3.63;47.23] |
| CpG2 | 2.50 | [0.95;3.52] | 8.07 | [3.55;52.63] |
| CpG3 | 2.68 | [1.36;3.50] | 6.02 | [3.51;46.94] |
| CpG4 | 2.63 | [1.49;3.33] | 7.06 | [3.35;52.17] |
| CpG5 | 3.41 | [1.61;4.56] | 10.12 | [4.72;49.32] |

**Supplementary Table S2.** Methylation level distribution for *NR3C1* CpG1, CpG3, CpG4 and CpG5 in those not bullied and those who reported bullying. The Kupol Study, Stockholm 2013-2015.

| **CpG site** | **Methylation level for individuals not bullied** | | **Methylation level for individuals bullied** | |
| --- | --- | --- | --- | --- |
| **Total number of samples**  **N = 1129** | Mean | Range [min;max] | Mean | Range [min;max] |
| CpG1 | 0.87 | [0;47.23] | 1.06 | [0;22.11] |
| CpG2 | 0.61 | [0;52.63] | 0.98 | [0;17.39] |
| CpG3 | 2.17 | [0;46.94] | 2.37 | [0;21.85] |
| CpG4 | 0.92 | [0;52.17] | 1.00 | [0;21.24] |
| CpG5 | 0.52 | [0;49.32] | 0.86 | [0;19.55] |

**Supplementary Table S3.** Methylation level distribution for *NR3C1* CpG1, CpG3, CpG4 and CpG5 in those with friends and those who reported lacking friends. The Kupol Study, Stockholm 2013-2015.

| **CpG site** | **Methylation level for individuals with friends** | | **Methylation level for individuals with no friends** | |
| --- | --- | --- | --- | --- |
| **Total number of samples**  **N = 1135** | Mean | Range [min;max] | Mean | Range [min;max] |
| CpG1 | 0.88 | [0;47.23] | 1.18 | [0;19.98] |
| CpG2 | 0.62 | [0;52.63] | 1.23 | [0;17.39] |
| CpG3 | 2.21 | [0;46.94] | 2.21 | [0;19.56] |
| CpG4 | 0.91 | [0;52.17] | 1.01 | [0;19.34] |
| CpG5 | 0.56 | [0;49.32] | 0.68 | [0;18.56] |

**Supplementary Table S4**. Cross-sectional Odds Ratio and corresponding 95% Confidence Interval for *NR3C1* CpG1, CpG3, CpG4 and CpG5 methylation according to potential stressors. The Kupol Study, Stockholm 2013-2015.

| **CpG1** | | | | | | |
| --- | --- | --- | --- | --- | --- | --- |
| **Potential stressors** | | **Proportion Non-methylated** | **Proportion Low methylated** | **OR_Low vs zero_, 95% CI** | **Proportion High methylated** | **OR_High vs zero_, 95% CI** |
| Bullying | Yes  N = 180 | 80.6% | 8.9% | 0.99,  0.56-1.74 | 10.5% | 1.26,  0.74-2.15 |
|  | No  N = 949 | 82.3% | 9.2% | Ref | 8.5% | Ref |
| Friends in school | No  N = 75 | 80% | 9.3% | 1.07,  0.48-2.41 | 10.7% | 1.25,  0.58-2.70 |
|  | Yes  N = 1061 | 82.3% | 8.9% | Ref | 8.8% | Ref |
| Currently using tobacco | Yes  N = 15 | 80.0% | 6.7% | 0.76,  0.10-5.92 | 13.3% | 1.55,  0.34-7.04 |
|  | No  N = 1134 | 82.2% | 9.0% | Ref | 8.8% | Ref |
| Alcohol the past year | Yes  N = 21 | 85.7% | 9.5% | 1.01,  0.23-4.41 | 4.8% | 0.51,  0.07-3.85 |
|  | No  N = 1117 | 82.0% | 9.0% | Ref | 9.0% | Ref |
| Both parents’ education below university | Yes  N = 315 | 80.3% | 10.5% | 1.27,  0.82-1.98 | 9.2% | 1.09,  0.69-1.72 |
|  | No  N = 827 | 82.0% | 9.0% | Ref | 9.0% | Ref |
| At least one parent unemployed | Yes  N = 196 | 82.7% | 9.7% | 1.07,  0.63-1.81 | 7.6% | 0.85,  0.48-1.50 |
|  | No  N = 936 | 87.6% | 6.5% | Ref | 5.9% | Ref |
| At least one parent born outside Sweden | Yes  N = 196 | 85.7% | 6.6% | 0.67,  0.37-1.23 | 7.7% | 0.80,  0.45-1.42 |
|  | No  N = 903 | 81.5% | 9.4% | Ref | 9.1% | Ref |
| Parents not cohabiting | Yes  N= 199 | 83.9% | 7.5% | 0.77,  0.43-1.36 | 8.6% | 0.95,  0.55-1.64 |
|  | No  N = 923 | 81.7% | 9.5% | Ref | 8.8% | Ref |

| **CpG3** | | | | | | |
| --- | --- | --- | --- | --- | --- | --- |
| **Potential stressors** | | **Proportion Non-methylated** | **Proportion Low methylated** | **OR_Low vs zero_, 95% CI** | **Proportion High methylated** | **OR_High vs zero_, 95% CI** |
| Bullying | Yes  N = 180 | 51.37% | 22.8% | 0.84,  0.56-1.25 | 25.5% | 0.96,  0.65-1.42 |
|  | No  N = 949 | 49.0% | 25.8% | Ref | 25.2% | Ref |
| Friends in school | No  N = 75 | 54.7% | 21.3% | 0.75,  0.41-1.37 | 24.0% | 0.85,  0.48-1.50 |
|  | Yes  N = 1061 | 49.1% | 25.5% | Ref | 25.4% | Ref |
| Currently using tobacco | Yes  N = 15 | 46.7% | 26.7% | 1.13,  0.33-3.88 | 26.6% | 1.12,  0.32-3.85 |
|  | No  N = 1134 | 49.6% | 25.1% | Ref | 25.3% | Ref |
| Alcohol the past year | Yes  N = 21 | 61.9% | 23.8% | 0.76,  0.27-2.14 | 14.3% | 0.44,  0.13-1.56 |
|  | No  N = 1117 | 49.2% | 25.1% | Ref | 25.7% | Ref |
| Both parents’ education below university | Yes  N = 315 | 49.2% | 25.8% | 0.98,  0.72-1.35 | 26.0% | 1.06,  0.77-1.45 |
|  | No  N = 827 | 49.7% | 25.4% | Ref | 24.9% | Ref |
| At least one parent unemployed | Yes  N = 196 | 54.1% | 24.5% | 0.87,  0.59-1.26 | 21.4% | 0.74,  0.50-1.10 |
|  | No  N = 936 | 48.6% | 25.4% | Ref | 26.0% | Ref |
| At least one parent born outside Sweden | Yes  N = 196 | 52.6% | 24.5% | 0.89,  0.61-1.30 | 23.0% | 0.83,  0.57-1.22 |
|  | No  N = 903 | 48.8% | 25.5% | Ref | 25.7% | Ref |
| Parents not cohabiting | Yes  N= 199 | 54.3% | 24.6% | 0.86,  0.59-1.25 | 21.1% | 0.72,  0.49-1.07 |
|  | No  N = 923 | 48.4% | 25.6% | Ref | 26.0% | Ref |

| **CpG4** | | | | | | |
| --- | --- | --- | --- | --- | --- | --- |
| **Potential stressors** | | **Proportion Non-methylated** | **Proportion Low methylated** | **OR_Low vs zero_, 95% CI** | **Proportion High methylated** | **OR_High vs zero_, 95% CI** |
| Bullying | Yes  N = 180 | 81.7% | 6.7% | 0.66,  0.35-1.23 | 11.6% | 1.24,  0.75-2.07 |
|  | No  N = 949 | 80.7% | 10.0% | Ref | 9.3% | Ref |
| Friends in school | No  N = 75 | 80.0% | 12.0% | 1.35,  0.65-2.80 | 8.0% | 0.85,  0.36-2.01 |
|  | Yes  N = 1061 | 81.3% | 9.1% | Ref | 9.6% | Ref |
| Currently using tobacco | Yes  N = 15 | 80.0% | 6.7% | 0.72,  0.09-5.62 | 13.3% | 1.43,  0.32-6.50 |
|  | No  N = 1134 | 81.2% | 9.4% | Ref | 9.4% | Ref |
| Alcohol the past year | Yes  N = 21 | 90.5% | 4.7% | 0.46,  0.06-3.50 | 4.8% | 0.45,  0.06-3.37 |
|  | No  N = 1117 | 81.2% | 9.2% | Ref | 9.6% | Ref |
| Both parents’ education below university | Yes  N = 315 | 80.3% | 8.9% | 0.97,  0.62-1.54 | 10.8% | 1.25,  0.81-1.92 |
|  | No  N = 827 | 81.9% | 9.3% | Ref | 8.8% | Ref |
| At least one parent unemployed | Yes  N = 196 | 79.1% | 9.7% | 1.11,  0.65-1.87 | 11.2% | 1.30,  0.79-2.14 |
|  | No  N = 936 | 81.9% | 9.1% | Ref | 9.0% | Ref |
| At least one parent born outside Sweden | Yes  N = 196 | 82.6% | 8.7% | 0.90,  0.52-1.56 | 8.7% | 0.89,  0.52-1.54 |
|  | No  N = 903 | 81.1% | 9.4% | Ref | 9.5% | Ref |
| Parents not cohabiting | Yes  N= 199 | 81.9% | 8.0% | 0.84,  0.48-1.46 | 10.1% | 1.08,  0.65-1.81 |
|  | No  N = 923 | 81.3% | 9.5% | Ref | 9.2% | Ref |

| **CpG5** | | | | | | |
| --- | --- | --- | --- | --- | --- | --- |
| **Potential stressors** | | **Proportion Non-methylated** | **Proportion Low methylated** | **OR_Low vs zero_, 95% CI** | **Proportion High methylated** | **OR_High vs zero_, 95% CI** |
| Bullying | Yes  N = 180 | 90.0% | 3.3% | 0.77,  0.32-1.83 | 6.7% | 1.79,  0.91-3.51 |
|  | No  N = 949 | 91.8% | 4.4% | Ref | 3.8% | Ref |
| Friends in school | No  N = 75 | 90.7% | 5.3% | 1.33,  0.46-3.82 | 4.0% | 0.95,  0.29-3.15 |
|  | Yes  N = 1061 | 91.7% | 4.1% | Ref | 4.2% | Ref |
| Currently using tobacco | Yes  N = 15 | 100% | 0.0% | ND | 0.0% | ND |
|  | No  N = 1134 | 91.5% | 4.2% | Ref | 4.3% | Ref |
| Alcohol the past year | Yes  N = 21 | 100% | 0.0% | ND | 0.0% | ND |
|  | No  N = 1117 | 91.4% | 4.3% | Ref | 4.3% | Ref |
| Both parents’ education below university | Yes  N = 315 | 90.4% | 4.8% | 1.25,  0.67-2.35 | 4.8% | 1.22,  0.65-2.27 |
|  | No  N = 827 | 92.1% | 3.9% | Ref | 4.0% | Ref |
| At least one parent unemployed | Yes  N = 196 | 91.3% | 4.6% | 1.14,  0.54-2.39 | 4.1% | 0.98,  0.45-2.14 |
|  | No  N = 936 | 91.8% | 4.0% | Ref | 4.2% | Ref |
| At least one parent born outside Sweden | Yes  N = 196 | 95.9% | 1.0% | 0.20,  0.05-0.84 | 3.1% | 0.65,  0.27-1.57 |
|  | No  N = 903 | 90.8% | 4.8% | Ref | 4.4% | Ref |
| Parents not cohabiting | Yes  N= 199 | 92.0% | 4.0% | 0.95,  0.44-2.06 | 4.0% | 0.97,  0.45-2.12 |
|  | No  N = 923 | 91.7% | 4.2% | Ref | 4.1% | Ref |
