# Supplementary figures and images for: NR3C1 hypermethylation in depressed and bullied adolescents
Source: Transl Psychiatry. 2018 Jun 19;8:121. doi: 10.1038/s41398-018-0169-8 (PMC6008402; doi:10.1038/s41398-018-0169-8)

**Supplementary Figure.**

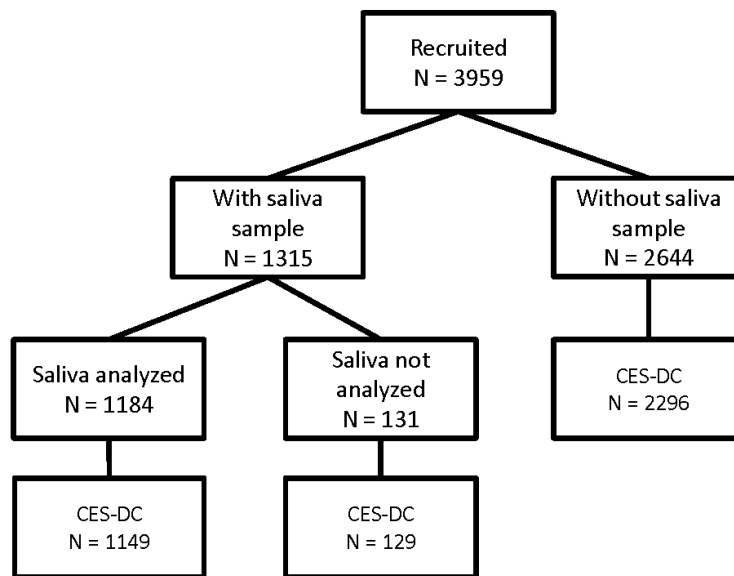

Flow chart of the participation. The Kupol Study, Stockholm 2013-2015.

Supplement: Supplementary file 2 — Supplementary figure [file 41398_2018_169_MOESM2_ESM.pdf]
